# Supplementary material for: Coral growth, survivorship and return-on-effort within nurseries at high-value sites on the Great Barrier Reef
Source: PLoS One. 2021 Jan 11;16(1):e0244961. doi: 10.1371/journal.pone.0244961 (PMC7799815; doi:10.1371/journal.pone.0244961)
Supplement: S1 Fig — (DOCX) [file pone.0244961.s001.docx]

**S1 Fig.** Examples of nursery platform design deployed at Opal Reef sites RayBan (RB) and Blue Lagoon (BL) – see main text – (images from RB, ca. 5m). Image on right shows routine cleaning from surgeonfish.

**
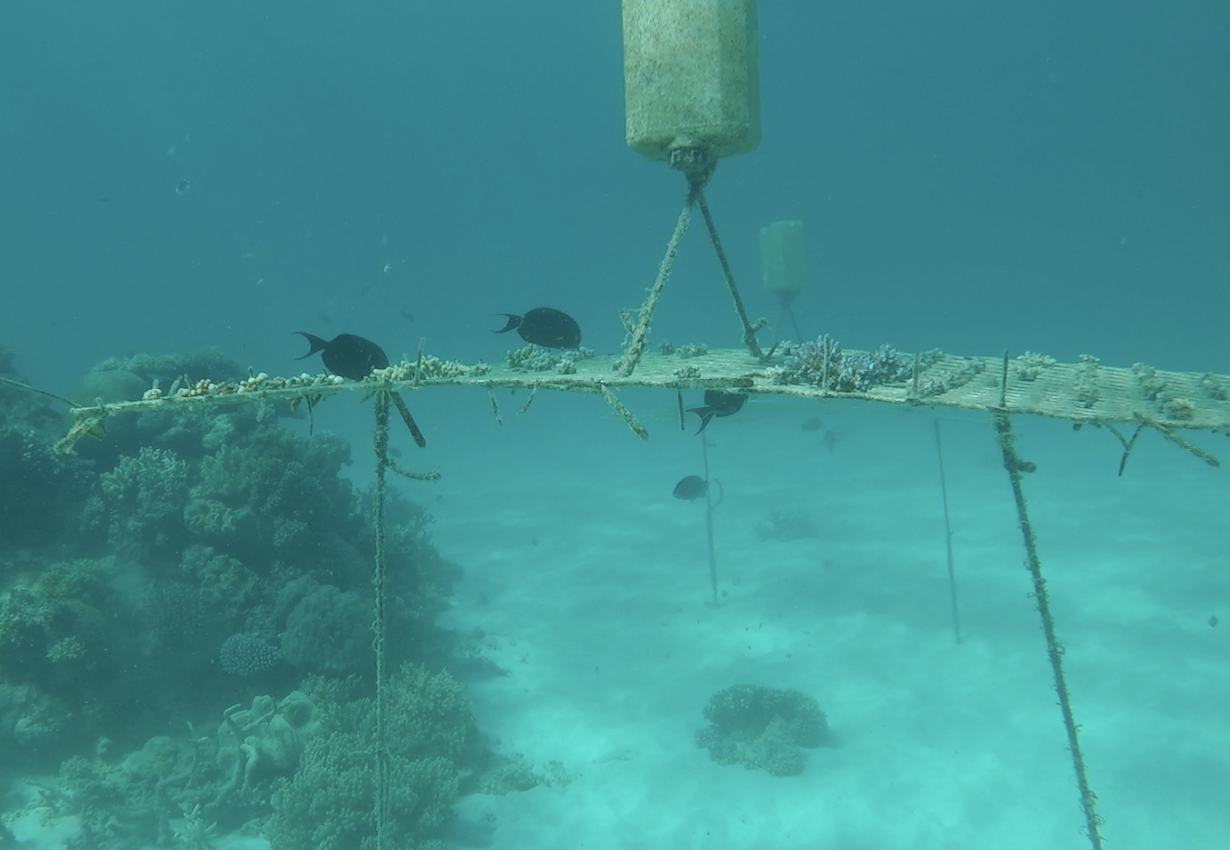

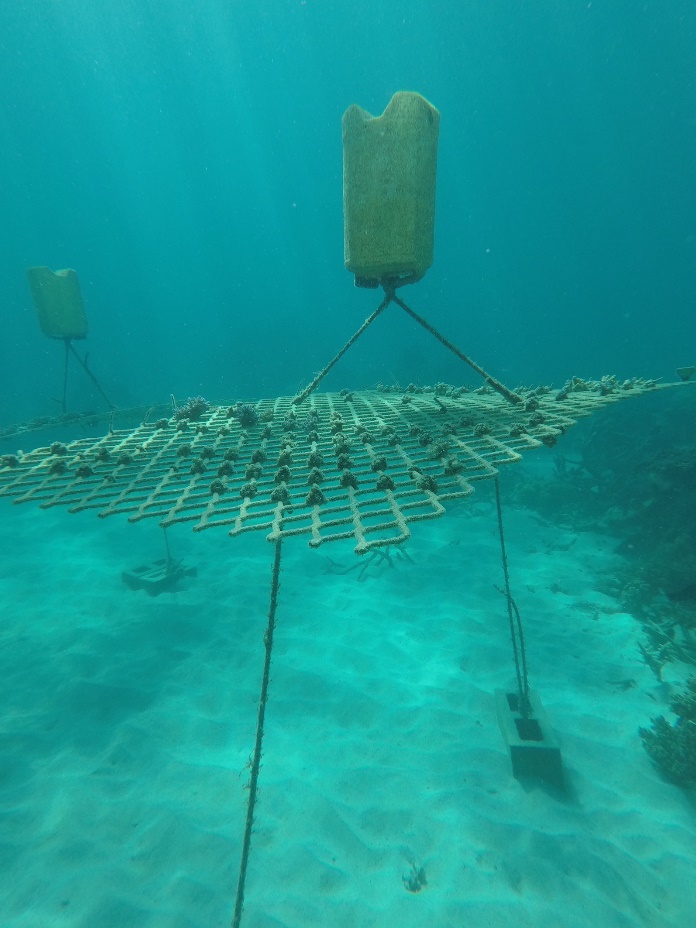
**
