# Supplementary material for: Coral growth, survivorship and return-on-effort within nurseries at high-value sites on the Great Barrier Reef
Source: PLoS One. 2021 Jan 11;16(1):e0244961. doi: 10.1371/journal.pone.0244961 (PMC7799815; doi:10.1371/journal.pone.0244961)
Supplement: S2 Fig — (DOCX) [file pone.0244961.s002.docx]

**S2** **Fig.** Percentage increase in coral growth (% month^-1^) areal extension (see methods) for eight coral species at the two nursery sites (RayBan, Blue Lagoon) at Opal Reef (*Acropora humilis*, A. hum; *Acropora hyacinthus*, A. hya; *Acropora loripes*; A. lor, *Acropora millepora*, A. mil; *Acropora tenuis*, A. ten; *Montipora hispida*, M. his; *Pocillopora cf. verrucosa*; *Porites cylindrica*, P. cyl).
