# Supplementary material for: Coral growth, survivorship and return-on-effort within nurseries at high-value sites on the Great Barrier Reef
Source: PLoS One. 2021 Jan 11;16(1):e0244961. doi: 10.1371/journal.pone.0244961 (PMC7799815; doi:10.1371/journal.pone.0244961)

**S3 Fig.** Comparative plots of (Ln transformed) %growth and (asin) transformed %survivorship – these 2D plots are used to then score return-on-effort (RRE) as per Suggett et al. (2019). Data compared are for (a) BL over the full year (August 2018-July 2019), (b) RB over the full year (May 2018-February 2018), (c) BL “cool season” only (August-December 2018) – see Figure 1 inset, and (d) BL “warm season” only (December 2018-July 2019).


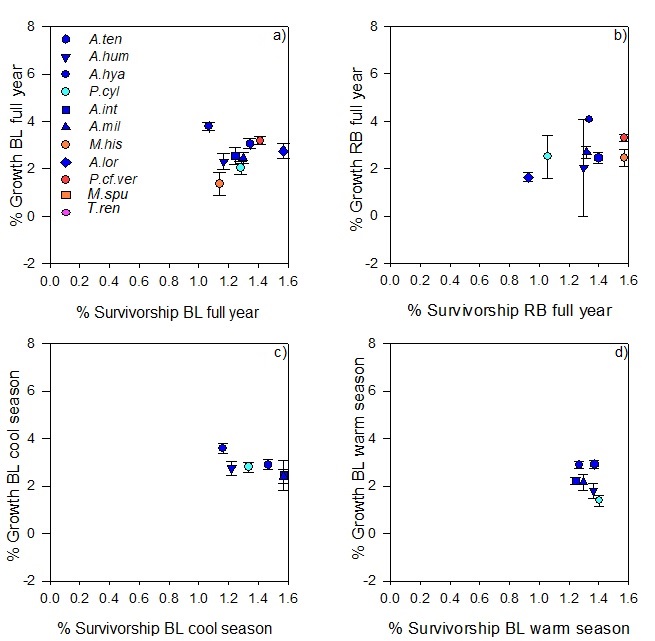

Supplement: S3 Fig — (DOCX) [file pone.0244961.s003.docx]
