# Supplementary material for: Coral growth, survivorship and return-on-effort within nurseries at high-value sites on the Great Barrier Reef
Source: PLoS One. 2021 Jan 11;16(1):e0244961. doi: 10.1371/journal.pone.0244961 (PMC7799815; doi:10.1371/journal.pone.0244961)
Supplement: S1 Table — (DOCX) [file pone.0244961.s004.docx]

**S1 Table.** Hard and total coral cover ($\pm$ standard error) at four Opal Reef sites: SNO, RayBan (RB), Blue Lagoon (BL), and Beautiful Mooring (BM), based on replicate (n=3) 30m continuous line intercept transects per site. Surveys conducted in October 2018. All transect data was collected and analysed – including binning into benthic categories – as per Gardner et al. 2019. Ecology & Evolution 9: 938-956.

| **Site** | **Total coral cover (%)** | **Hard coral cover (%)** |
| --- | --- | --- |
| SNO | 24.1 (9.0) | 22.6 (8.9) |
| RB | 31.1 (3.6) | 17.1 (1.7) |
| BL | 43.2 (2.1) | 39.8 (2.2) |
| BM | 40.4 (3.4) | 35.1 (5.4) |
