# Supplementary material for: Coral growth, survivorship and return-on-effort within nurseries at high-value sites on the Great Barrier Reef
Source: PLoS One. 2021 Jan 11;16(1):e0244961. doi: 10.1371/journal.pone.0244961 (PMC7799815; doi:10.1371/journal.pone.0244961)
Supplement: S2 Table — (DOCX) [file pone.0244961.s005.docx]

**S2 Table.** ANOVA and *post hoc* Tukey Tests (p<0.05) of: (i) Absolute growth (cm^2^ month^-1^), (ii) % Growth (Ln transformed) month^-1^ and (iii) return-on-effort (RRE), at BL (August 2018-July 2019) binned by species. Values that were not significantly different from one another are grouped within square brackets; each set of square brackets indicates significantly different groups of species while hyphens indicate overlapping groupings. Statistical analysis was conducted using R Studio version 1.1.423 (RStudio Team 2015). Test for normality (qq-plots) and equal variance (Levene’s test) were passed.

| **Test** | **Group** | **F** | ***p*** | ***Post hoc* approach groupings**  **(*p*< 0.05)** |
| --- | --- | --- | --- | --- |
| Absolute growth | Species (BL) | 22.37 | <0.001 | **Ahya-Ahum**  Aint-Ahum  Alor-Ahum  Amill-Ahum  **Aten-Ahum**  Mhis-Ahum  Pcyl-Ahum  Pver-Ahum  **Aint-Ahya**  **Alor-Ahya**  **Amill-Ahya**  **Aten-Ahya**  **Mhis-Ahya**  **Pcyl-Ahya**  **Pver-Ahya**  Alor-Aint  Amill-Aint  Aten-Aint  Mhis-Aint  Pcyl-Aint  Pver-Aint  Amill-Alor  Aten-Alor  Mhis-Alor  Pcyl-Alor  Pver-Alor  **Aten-Amill**  Mhis-Amill  Pcyl-Amill  Pver-Amill  Mhis-Aten  **Pcyl-Aten**  Pver-Aten  Pcyl-Mhis  Pver-Mhis  Pver-Pcyl |
| % Growth/month | Species (BL) | 5.45 | <0.001 | [A.hya]-[A.int, A.lor, A.ten, P.ver]-A.hum, A.mil., M.his, P.cyl] |
| RRE | Species (BL) | 8.38 | <0.001 | [A.lor, A.ten, P.ver]-[A.mil]-[A.hum, A.hya]-[A.int, M.his, P.cyl] |
