# Supplementary material for: Coral growth, survivorship and return-on-effort within nurseries at high-value sites on the Great Barrier Reef
Source: PLoS One. 2021 Jan 11;16(1):e0244961. doi: 10.1371/journal.pone.0244961 (PMC7799815; doi:10.1371/journal.pone.0244961)
Supplement: S3 Table — (DOCX) [file pone.0244961.s006.docx]

**S3** **Table.** Two-way ANOVA and *post hoc* Tukey Tests (p<0.05) of: (i) Absolute growth (cm^2^ month^-1^), (ii) % Growth (Ln transformed) month^-1^ and (iii) return-on-effort (RRE), binned by site (BL versus RB) and by species (see Table S1). Statistical analysis was conducted using R Studio version 1.1.423 (RStudio Team 2015). Test for normality (qq-plots) and equal variance (Levene’s test) were passed. NS denotes non-significance.

| **Test** | **Group** | **F** | ***p*** | ***Post hoc* approach groupings (p<0.05)** |
| --- | --- | --- | --- | --- |
| Absolute growth | Species  Site  Species x site | 22.71  25.22  3.15 | <0.001  <0.001  0.003 | **Ahya-Ahum**  Alor-Ahum  Amill-Ahum  **Aten-Ahum**  Mhis-Ahum  Pcyl-Ahum  Pver-Ahum  **Alor-Ahya**  **Amill-Ahya**  **Aten-Ahya**  **Mhis-Ahya**  **Pcyl-Ahya**  **Pver-Ahya**  Amill-Alor  **Aten-Alor**  Mhis-Alor  Pcyl-Alor  **Pver-Alor**  **Aten-Amill**  Mhis-Amill  Pcyl-Amill  **Pver-Amill**  **Mhis-Aten**  **Pcyl-Aten**  Pver-Aten  Pcyl-Mhis  Pver-Mhis  Pver-Pcyl  BL-RB  **Ahya:BL-Ahum:BL**  Alor:BL-Ahum:BL  Amill:BL-Ahum:BL  **Aten:BL-Ahum:BL**  Mhis:BL-Ahum:BL  Pcyl:BL-Ahum:BL  Pver:BL-Ahum:BL  Ahum:RB-Ahum:BL  Ahya:RB-Ahum:BL  Alor:RB-Ahum:BL  Amill:RB-Ahum:BL  Aten:RB-Ahum:BL  Mhis:RB-Ahum:BL  Pcyl:RB-Ahum:BL  Pver:RB-Ahum:BL  **Alor:BL-Ahya:BL**  **Amill:BL-Ahya:BL**  **Aten:BL-Ahya:BL**  **Mhis:BL-Ahya:BL**  **Pcyl:BL-Ahya:BL**  **Pver:BL-Ahya:BL**  **Ahum:RB-Ahya:BL**  **Ahya:RB-Ahya:BL**  **Alor:RB-Ahya:BL**  **Amill:RB-Ahya:BL**  **Aten:RB-Ahya:BL**  **Mhis:RB-Ahya:BL**  **Pcyl:RB-Ahya:BL**  **Pver:RB-Ahya:BL**  Amill:BL-Alor:BL  Aten:BL-Alor:BL  Mhis:BL-Alor:BL  Pcyl:BL-Alor:BL  Pver:BL-Alor:BL  Ahum:RB-Alor:BL  Ahya:RB-Alor:BL  Alor:RB-Alor:BL  Amill:RB-Alor:BL  Aten:RB-Alor:BL  Mhis:RB-Alor:BL  Pcyl:RB-Alor:BL  Pver:RB-Alor:BL  Aten:BL-Amill:BL  Mhis:BL-Amill:BL  Pcyl:BL-Amill:BL  Pver:BL-Amill:BL  Ahum:RB-Amill:BL  Ahya:RB-Amill:BL  Alor:RB-Amill:BL  Amill:RB-Amill:BL  Aten:RB-Amill:BL  Mhis:RB-Amill:BL  Pcyl:RB-Amill:BL  Pver:RB-Amill:BL  **Mhis:BL-Aten:BL**  **Pcyl:BL-Aten:BL**  Pver:BL-Aten:BL  **Ahum:RB-Aten:BL**  Ahya:RB-Aten:BL  **Alor:RB-Aten:BL**  **Amill:RB-Aten:BL**  **Aten:RB-Aten:BL**  **Mhis:RB-Aten:BL**  Pcyl:RB-Aten:BL  Pver:RB-Aten:BL  Pcyl:BL-Mhis:BL  Pver:BL-Mhis:BL  Ahum:RB-Mhis:BL  Ahya:RB-Mhis:BL  Alor:RB-Mhis:BL  Amill:RB-Mhis:BL  Aten:RB-Mhis:BL  Mhis:RB-Mhis:BL  Pcyl:RB-Mhis:BL  Pver:RB-Mhis:BL  Pver:BL-Pcyl:BL  Ahum:RB-Pcyl:BL  Ahya:RB-Pcyl:BL  Alor:RB-Pcyl:BL  Amill:RB-Pcyl:BL  Aten:RB-Pcyl:BL  Mhis:RB-Pcyl:BL  Pcyl:RB-Pcyl:BL  Pver:RB-Pcyl:BL  Ahum:RB-Pver:BL  Ahya:RB-Pver:BL  **Alor:RB-Pver:BL**  **Amill:RB-Pver:BL**  Aten:RB-Pver:BL  Mhis:RB-Pver:BL  Pcyl:RB-Pver:BL  Pver:RB-Pver:BL  Ahya:RB-Ahum:RB  Alor:RB-Ahum:RB  Amill:RB-Ahum:RB  Aten:RB-Ahum:RB  Mhis:RB-Ahum:RB  Pcyl:RB-Ahum:RB  Pver:RB-Ahum:RB  Alor:RB-Ahya:RB  Amill:RB-Ahya:RB  Aten:RB-Ahya:RB  Mhis:RB-Ahya:RB  Pcyl:RB-Ahya:RB  Pver:RB-Ahya:RB  Amill:RB-Alor:RB  Aten:RB-Alor:RB  Mhis:RB-Alor:RB  Pcyl:RB-Alor:RB  Pver:RB-Alor:RB  Aten:RB-Amill:RB  Mhis:RB-Amill:RB  Pcyl:RB-Amill:RB  Pver:RB-Amill:RB  Mhis:RB-Aten:RB  Pcyl:RB-Aten:RB  Pver:RB-Aten:RB  Pcyl:RB-Mhis:RB  Pver:RB-Mhis:RB  Pver:RB-Pcyl:RB |
| % Growth month^-1^ | Species  Site  Species x site | 10.65  0.332  9.847 | <0.001  NS  NS | [A.hya] - [A.ten] - [A.lor]-[P.ver]-[A.hum, A.mil, M.his., P.cyl]  NS  NS |
| RRE | Species  Site  Species x site | 30.09  1.494  23.89 | <0.001  NS  <0.001 | [A.ten, P.ver]-[A.mil, M.his]-[A.hys, A.hum, P.cyl] - [A.lor]  NS  Alor:BL-Ahum:BL  Aten:BL-Ahum:BL  Pver:BL-Ahum:BL  Ahya:RB-Ahum:BL  Alor:RB-Ahum:BL  Amill:RB-Ahum:BL  Mhis:RB-Ahum:BL  Pver:RB-Ahum:BL  Alor:BL-Ahya:BL  Aten:BL-Ahya:BL  Pver:BL-Ahya:BL  **Ahya:RB-Ahya:BL**  Alor:RB-Ahya:BL  Mhis:RB-Ahya:BL  Pver:RB-Ahya:BL  Mhis:BL-Alor:BL  Pcyl:BL-Alor:BL  Ahum:RB-Alor:BL  **Alor:RB-Alor:BL**  Pcyl:RB-Alor:BL  Alor:RB-Amill:BL  Pver:RB-Amill:BL  Mhis:BL-Aten:BL  Alor:RB-Aten:BL  Pcyl:RB-Aten:BL  Pver:BL-Mhis:BL  Ahya:RB-Mhis:BL  Amill:RB-Mhis:BL  **Mhis:RB-Mhis:BL**  Pver:RB-Mhis:BL  Alor:RB-Pcyl:BL  **Pcyl:RB-Pcyl:BL**  Pver:RB-Pcyl:BL  Alor:RB-Pver:BL  Pcyl:RB-Pver:BL  Alor:RB-Ahum:RB  Pver:RB-Ahum:RB  Alor:RB-Ahya:RB  Pcyl:RB-Ahya:RB  Amill:RB-Alor:RB  Aten:RB-Alor:RB  Mhis:RB-Alor:RB  Pver:RB-Alor:RB  Pcyl:RB-Amill:RB  Pcyl:RB-Mhis:RB  Pver:RB-Pcyl:RB |
