# Supplementary material for: Coral growth, survivorship and return-on-effort within nurseries at high-value sites on the Great Barrier Reef
Source: PLoS One. 2021 Jan 11;16(1):e0244961. doi: 10.1371/journal.pone.0244961 (PMC7799815; doi:10.1371/journal.pone.0244961)
Supplement: S4 Table — (DOCX) [file pone.0244961.s007.docx]

**S4 Table.** Two-way ANOVA and *post hoc* Tukey Tests (p<0.05) of: (i) Absolute growth (cm^2^ month^-1^), (ii) % Growth (Ln transformed) month^-1^ and (iii) return-on-effort (RRE), at BL (August 2018-July 2019) binned by species (see Table S2) and by “season” (“warm” versus “cool”). Statistical analysis was conducted using R Studio version 1.1.423 (RStudio Team 2015). Test for normality (qq-plots) and equal variance (Levene’s test) were passed. NS denotes non-significance.

| **Test** | **Group** | **F** | ***p*** | ***Post hoc* approach groupings (p<0.05)** |
| --- | --- | --- | --- | --- |
| Absolute growth | Species  “Season”  Species x season | 18.70  0.64  1.34 | <0.001  NS  NS | **Ahya-Ahum**  Aint-Ahum  Amill-Ahum  **Aten-Ahum**  Pcyl-Ahum  **Aint-Ahya**  **Amill-Ahya**  **Aten-Ahya**  **Pcyl-Ahya**  Amill-Aint  Aten-Aint  Pcyl-Aint  **Aten-Amill**  Pcyl-Amill  **Pcyl-Aten**  NS  NS |
| % Growth/month | Species  “Season”  Species x season | 6.267  0.332  9.847 | <0.001  NS  <0.001 | [A.hya]-[A.int]-[A.hum, A.mil A.ten, P.cyl]  NS  Pcyl:warm-Ahum:cool  Aten:cool-Ahya:cool  Ahum:warm-Ahya:cool  Amill:warm-Ahya:cool  Pcyl:warm-Ahya:cool  Pcyl:cool-Aten:cool  Ahya:warm-Aten:cool  **Aten:warm-Aten:cool**  Aten:warm-Ahum:warm  Pcyl:warm-Ahya:warm  Pcyl:warm-Aten:warm |
| RRE | Species  “Season”  Species x season | 5.945  1.449  5.738 | <0.001  NS  <0.001 | [A.hum]-[A.int, P.cyl]-[A.hya, A.mil, A.ten]  NS  Amill:cool-Ahum:cool  Ahya:warm-Ahum:cool  Aten:warm-Ahum:cool  Ahum:warm-Amill:cool  Aint:warm-Amill:cool  Pcyl:warm-Amill:cool  Aten:warm-Ahum:warm  Aint:warm-Ahya:warm  Pcyl:warm-Ahya:warm  Aten:warm-Aint:warm  Pcyl:warm-Aten:warm |
