# Supplementary material for: Coral growth, survivorship and return-on-effort within nurseries at high-value sites on the Great Barrier Reef
Source: PLoS One. 2021 Jan 11;16(1):e0244961. doi: 10.1371/journal.pone.0244961 (PMC7799815; doi:10.1371/journal.pone.0244961)
Supplement: Attachment — Submitted filename: PLOS ONE response letter.docx [file pone.0244961.s009.docx]

**RESPONSE LETTER**

**Manuscript ID number:** PONE-D-20-11054

**Manuscript title:** Coral growth, survivorship and return-on-effort within nurseries at high-value sites on the Great Barrier Reef

**Author(s):** Lorna Howlett, Emma F. Camp, John Edmondson, Nicola Henderson, David. J. Suggett

Dear Editor in Chief,	8^th^ July 2020

We thank you and the Reviewers for the highly constructive feedback of our manuscript entitled *Coral growth, survivorship and return-on-effort within nurseries at high-value sites on the Great Barrier Reef*; the comments have helped strengthen our manuscript. We have considered all of the suggested edits and have made appropriate changes to the manuscript. These changes are documented in the table below as well as in the track-changed version of the updated manuscript. We have also thoroughly re-proofed the manuscript for any editorial errors. We hope the revised manuscript can now be accepted in PLOS One. All documented edits are track changed in the upload word document and have been outlined below alongside each Editor and Reviewer comment.

Kind Regards,

Dr Emma Camp, on behalf of all co-authors

| Reviewer & comment no. | Reviewer comment | Line no. in original ms | Line no. in revised ms | Author comment |
| --- | --- | --- | --- | --- |
|  | We thank the Editor and Reviewers 1 and 2 for their positive, reflective and fair review of our manuscript. We appreciate their suggestions that we have incorporated them to improve our manuscript. | | | |
| Editor 1 | Some of the first restoration interventions were aimed at addressing damage from ship-groundings in the Western Atlantic (Florida - see work by Harold Hudson, Bill Precht and others). These predated efforts in response to disease. | Lines 26-30 | Lines 31-36 | This example of previous coral restoration efforts has been added to the ms. |
| E2 | There are also approaches that provide stable substrate to allow for natural recovery via coral recruitment (artificial reefs) or those that restore environmental conditions so that corals can recover (passive restoration) | Line 35 | Lines 28-31 | Other passive and active restoration practices have been mentioned in the Introduction. |
| E3 | How were collected fragments kept while being processed on deck? Was there any exposure to air? Shading? A bit more detail on handling would be useful. | Line 143 | Lines 150-153 | More information on coral handling on-deck has been added to the ms. |
| E4 | Please use numbers in square brackets instead of year of publication for in-text references. | Line 270 | Line 204 | In-text reference has been replaced with “[26]” |
| E5 | *A. formosa* - use the junior synonym, *A. muricata*, throughout | Line 309 | Throughout | All references of *A. formosa* have been changed to *A. muricata*. |
| E6 | Please use numbers in square brackets instead of year of publication for in-text references. | Line 330 | Line 320 | In-text reference has been replaced with “[16]” |
| Reviewer 1.1 | The authors need to be very clear that this coral reef restoration attempt only covers the first phase of the coral gardening method which is the growing of coral fragments in nurseries. | N/A | Line 21 and throughout | Minor changes have been made to the abstract and introduction to further highlight that the ms focusses on the nursery phase of coral gardening. |
| 1.2 | It is somewhat difficult to compare the results from both sites which each other because both nurseries were running for different periods of time and  the authors should standardise the period of experiment before they make any comparisons. | N/A | N/A | Whilst both nurseries were initiated at slightly different times (and data collected at different months, we have ensured that we are transparent about the slightly different time frames for the two sites (methods) and how they relate to seasonal temperature (discussion). Importantly, all growth rates were standardised to cm^2^/month for direct inter-comparison. Our ANOVA results further demonstrated no significant differences between seasons for the site with an entire years’ worth of data, suggesting as we discuss – that the different time frames of observations between sites is likely insignificant. |
| 1.3 | I’d like to see some recommendations of management of the area or how the approach could be scaled-up. | N/A | Lines 416-418 | We are mindful of over-stretching how the nurseries could aid management – sure, scaling up should be straightforward assuming that costs remain low where adopted by other stewards. To complement the existing text in the conclusions that describe this point, we have added the text “Furthermore, assessing how well such nursery infrastructure can be adopted by other Great Barrier Reef site stewards will be critical to fully resolve the utility and effectiveness as a low-cost site management aid.”. |
| 1.4 | I'd like to know whether there were any considerations with respect to genetic diversity prior to selecting the coral donors. | N/A | Lines 144-146 | Unfortunately, we have no knowledge of genetic diversity (or of how colonies disperse) for many of the species on the Great Barrier Reef, in particular at local reef scales such as in our current study (Opal Reef). This is the focus of future work (and will be a large study in its own right which is beyond the scope of the present study) to understand how genetic variation within species are dispersed (and how they align to different phenotypes). As we cannot comment on the genetic diversity inherent to our study, we have included the additional line in the methods “Source colonies for any given species were sampled randomly throughout the local population (spaced at least 5m from one another).” |
| 1.5 | Please defer from using abbreviations such as RB, BL, RRE and delta G in the abstract. The abstract does not explain how the return-on-effort scores are being calculated or what they mean. Be clear that the study captures only the coral nursery phase of the coral gardening approach and not the outplanting phase. | Abstract, lines 2-22 | Lines 2-25 | All abbreviations have been removed from the abstract.  The merit for using RRE has been given in the Abstract, and more information on its use alongside analyses of growth and survivorship in isolation of one another has been given in the main text.  Additions have been made to the Abstract to highlight that the study only captures the initial nursery phase of “coral gardening”. |
| 1.6 | Recalculate the growth rates after 9 months for both locations, so that a direct comparison between both is possible over the same period of time. | Line 11 | N/A | Repeated comment – see 1.2 |
| 1.7 | Please write the exact number of coral fragments in the nursery. Using ‘ca.’ is confusing. | Line 24 | Lines 14-15 | Exact number now provided |
| 1.8 | This part belongs in the discussion section. | Lines 52-55 | Discussion | This part has been moved to the Discussion section. |
| 1.9 | If the nursery has been located in the Conservation Park Zone, the authors should provide permit + permit number that they must have received from GBRMPA or clarify that the restoration site was not within the permit area. I saw – this comes later in LN143. | Line 91 | N/A | As pointed out by Reviewer 1, the permit number is given in line 143 (now line 275). The permit number is also stated in the Acknowledgements. |
| Reviewer 2.1 | While the growth and survivorship results are straight forward, there is little justification for using RRE | N/A | Lines 81-85 | We have now modified the text at the end of the introduction to clarify the justification – whereby the RRE score compliments assessments of growth and survivorship in isolation of one another; specifically “As such the RRE compliments assessment of examining growth and survivorship in isolation of one another as “measures of success” to consider how growth and survivorship interact for any given species, e.g. differences in species with “r versus k” life history strategies that carry inherent trade-offs in growth rates and stress tolerance [26].” |
| 2.2 | Little evidence was presented that restoration was needed at the site. | N/A | See replies 2.8 and 2.15 | This is addressed in response to Reviewer 2’s comments 2.8 and 2.15 below. |
| 2.3 | The statistics used were overkill for what was a straight-forward study. Sometimes less is more. There is lots of redundancy in the Tables and Figures, along with repetition in the narrative. | N/A | Table and Figure captions | Excessive narrative has been removed from Table and Figure captions. |
| 2.4 | I did not find that the supplemental figures added to the story, no raw data in supplementary materials. | Supplementary Materials | Supplementary Materials | The raw data has been added to the Supplementary Materials. |
| 2.5 | I found the combination of Results and Discussion awkward. | N/A | Results and Discussion | The combined Results & Discussion section has been separated into a Results section and a Discussion section. |
| 2.6 | The use of e.g. and other qualifiers attached to references are not needed. In general, I didn’t try to copy edit. | Line 28 and throughout | Throughout | All uses of “e.g.” and “see” before references have been removed from the ms. |
| 2.7 | Need is now greater based on recent loss of coral because of global warming in 2016/17. Heat waves is a euphemism that could be interpreted as natural events. | Line 31 | Lines 38-39 | This sentence has been changed. |
| 2.8 | Outplanting is conducted in Florida and the Caribbean because recruitment does not occur, or rarely occurs. The authors should provide examples of where recruitment is so low that it impacts “high-value sites.” | Line 36 | Lies 39-41 | We now state in the final line of the first paragraph of the introduction “Many GBR “high-value reef sites”…face reduced coral abundance and rates of natural recovery [13].” Importantly, reference 13 captures many northern GBR reefs sites – including Opal Reef that has indicated reduced recruitment (with a shift to brooders). |
| 2.9 | Semi-continuously = continually | Line 38 | Line 45 | This has been altered in the ms. |
| 2.10 | re- (or out-) planting = outplanting or transplanting (pick one then be consistent throughout paper). | Line 42 | Throughout | All mentions of “out-planting” and “re-planting” have been changed to “outplanting”. |
| 2.11 | The premise that the variety of approaches result in variable results is not supported. Alternatively, the variety of results is a result of what the authors list as different environmental conditions, alternative coral species and genotypes. The point is that nursery methods generally work well, no matter the technique used to grow the corals. Rationale for the work is overstated by saying uncertainty constrains the capacity to effectively grow corals in nurseries. Methods to grow branching corals are well known. Methods to growth boulder or mounding corals are advancing. | Line 52 | Lines 58-69 | We apologise – this inference “the work is overstated by saying uncertainty constrains the capacity to effectively grow corals in nurseries” was not our intent. We agree that nurseries work well (and state “species propagated within nurseries appear to exhibit growth rates and survivorship similar to, or exceeding, those of source colonies within natural reef habitats”). The subsequent text at the end of this paragraph was to state that rarely have systematic studies been conducted to isolate the effect of different nursery types, coral species or environments affect the ultimately gain nurseries can return. We have therefore edited the final sentence of this paragraph for clarity. |
| 2.12 | There is debate about using naturally occurring fragments for outplanting because with sites that are depauperate of corals you can end up in a zero-sum situation. Obviously, if there is plenty of source material then collection for fragments isn’t a problem. But if there’s plenty of source material, why restore? | Line 66 | N/A | We disagree with the reviewer here. There is not “plenty” of source material but understand the need to better justify the need to “restore” (see reply to comment 2.15). We did not understand the comment on the ‘zero-sum’ situation since many fragments of opportunity typically fall on sand and die at Great Barrier Reef sites, and so re-planting these (and preventing mortality) surely is a sum gain? We refer to this point in the introduction by citing the paper where we have recently discussed this [27]. |
| 2.13 | Condition (percent living tissue) is an important metric can affect growth and survivorship, but is not discussed (or included in the study). | Line 75 | Lines 186-189 | We agree with the Reviewer that this was not directly measured; however, this reflected our sampling approach of removing any fragments from the nursery with tissue loss – we appreciate this was not explicit in the methods and have now added the lines “Furthermore, any fragments with visible signs of tissue loss were removed (as per permitting conditions to pre-empt potential disease outbreaks) and accounted for as “loss” under survivorship; as such, any size measurements inherently contained 100% tissue cover.” |
| 2.14 | The authors cite high growth, but they need to place this introductory statement in context to corals from the wild. As stated, the reader is left to assume what high growth means. All corals in both nurseries? | Line 75 | Lines 85-89 | We state on line 59, “species propagated within nurseries appear to exhibit growth rates and survivorship similar to, or exceeding, those of source colonies within natural reef habitats [10, 15, 20, 22, 24].”  We have now clarified the final sentence to read “In doing so, we show for the first time how nurseries can yield variable growth rates of 0.6-10.8 cm^2^month^-1^ and high survivorship of > 80% at two frequently visited tourism (high-value) sites on the GBR…”– we agree with the reviewer that “benchmarking” what we mean by high is important. |
| 2.15 | The Introduction cites justification for the nursery and I assume eventual outplanting because of coral mortality caused by 2016/17 coral bleaching, but the authors state here that the “exact impact from the 2016/17 coral bleaching event is unknown.” Instead, they site a Pers Obs that mortality was patchy. Also, later (L149) they state that species were chosen for their relatively high abundance. That would seem to diminish the need for restoration. | Line 95 | Lines 103-107 | The Reviewer is correct that the original motivation for coral propagation work at Opal Reef was in response to cumulative degradation from repeat bleaching events (as we state in the text). However, we have now clarified the extent of this decline in the main text “Whilst manta tow surveys around Opal Reef suggest that the 2016/17 coral bleaching event reduced hard coral cover from 21% (2015) to 8% (2019) [28] ” and include results of benthic surveys.  The fact species abundance is relatively high does not mean total cover is high (and hence there is a very real need for site rehabilitation) – we have clarified this sentence however to read “…given their relatively high abundance within the total coral cover…” |
| 2.16 | What is the difference between targeted and localized restoration? | Line 95 | Line 108 | “Targeted” has been removed from this sentence to avoid repetition. |
| 2.17 | What is a “key site,” versus a regular site for the installation of a nursery? | Line 95 | Line 109 | The word “key” has been removed from this sentence to avoid overstating the significance of chosen sites. |
| 2.18 | Fig 1 Discussion of SST appears in the narrative of this section and the figure caption. Drop in figure caption. | Line 124 | Line 118 | This narrative has been removed from the figure caption. |
| 2.19 | Corals were held at 4-6 m depth on the frames, stated as same depth as from where corals were sourced. That makes sense for RB but not BL that is stated as 8 m depth. Corals could be 4 m shallower on the frames at BL if collected from 8 m, the stated depth of the site. | Line 138 | Lines 139-141 | This line has been changed to read “frames were suspended approximately 1-2m above the substrate to reduce potential sedimentation so that corals were held at 4-5m and 6-7m depth for RB and BL nurseries, respectively”. We hope this has made the statement easier to understand. |
| 2.20 | Authors state that a random subset of corals was monitored for fate-tracking. This is confusing. It appears that a subset was monitored for growth but all corals were monitored for survivorship. | Line 156 | Lines 165-169 | The ms states that a subset was monitored for fate-tracking growth and all of the fragments were monitored for survival. We believe the statement is clear and easy to understand. |
| 2.21 | With different numbers of fragments to start per species why does it make sense to randomly select among all fragments for fate tracking? | Line 156 | N/A | Repeated comment – see 2.20 |
| 2.22 | Seeding was conducted in May and August 2018, yet earlier it was stated that RB was installed in February (line 118). | Line 173 | Lines 141-142 | The Reviewer is correct, frames were installed earlier than seeding to enable time for frame conditioning (as we state elsewhere in the text). We have altered this sentence to read “All aluminium frames were conditioned *in situ* for a period of at least 2 weeks prior to seeding with coral”. |
| 2.23 | Why would you want to evaluate success as the outcome of growth versus survivorship using RRE? No rationale is provided. Indeed. RRE obscures important information about performance in the nursery because ultimately you want larger numbers and larger fragments that are in good condition. | Line 191 | Lines 202-205 | We have now clarified the complimentary use of RRE in the Introduction (see reply to comment 2.1) – however, we have also clarified in the methods here to read “In order to further evaluate success, but from any life-history strategy trade-off of growth versus survivorship as recently detailed…” – in doing so we are clear that the goal is not to obscure information (since we first look at growth and survivorship as mutually exclusive variables) but rather that the RRE metric is a subsequent means to consider the trade-off of the two variables. |
| 2.24 | At some point, size can become problematic because fragmentation can occur or the corals can start growing into each other or the weight can sink the frames. This seems to be a metric is search of a problem. The condition (percent live tissue) also matters, but it was not measured. | Line 191 | See reply to Reviewer 2.13 point. | The Reviewer makes an interesting observation with regards to the future need to solve this problem. We have addressed the comment on live tissue above (Point 2.13) |
| 2.25 | I generally prefer that Results and Discussion are presented separately. That way, the significance and context of the work relative to other studies are presented in one location – the Discussion. As presented, it’s difficult to find the story. | Line 211 | Results and Discussion | Repeated comment - see 2.5 (sections now separated). |
| 2.26 | Growth rates within GBR nurseries (Two nurseries at one reef is not the GBR) | Line 211 | N/A | We are unsure which line in the Results and Discussion section this is referring to. We have taken precautions to ensure the significance of the results have not been overstated. |
| 2.27 | Be consistent throughout the paper in the order that information about the two nurseries is presented. In Materials and Methods RB is presented first, then BL. So present RB results first, then BL, then compare them. | Line 213 | Results section | We prefer to report the most relevant results first, rather than simply stating them in order of first mention. |
| 2.28 | Generally, there is an excessive amount of narrative that is also found in Tables 2, 3 and Fig 2. | Tables 2, 3 and Fig 2 | All Tables and Figures | Any non-essential narrative has been removed from all table and figure captions in the ms. |
| 2.29 | It would be useful to include growth data in Table 2 from wild populations. That way, comparisons with the fragments are all in one place and not scattered in the narrative. | Table 2 | See reply to comment 2.14 | Unfortunately, we do not have growth rate data form wild (source) colonies for this study – as we state in reply to comment 2.14. We appreciate that absolute growth rates from source colonies on the reef is important to determine in future. |
| 2.30 | RRE score could be a separate table, but I find the metric useless and the paper would not suffer if it was dropped. | Table 2 | See replies to comments 2.1 and 2.23 | We disagree – we have clarified the justification and value in RRE as a metric that compliments assessments of growth and survivorship (replies to comments 2.1 and 2.23). |
| 2.31 | If survivorship curves are routinely used to track nursery coals, then citations would be useful. They are absent. | Results & Discussion | Line 364 | “Survivorship curves” has been changed to “survivorship” and a reference has been given for this statement. |
| 2.32 | RRE values are presented in detail, but their relevance is not clear. Why is it useful to calculate this metric? What do you get from RRE that is absent from the growth and survivorship data? How can RRE be used to “optimize any given practice?” | N/A | See replies to comments 2.1 and 2.23 | Repeated comment - see comment 2.1 and 2.23 |
| 2.33 | The discussion and justification of RRE as a useful metric that appears in lines 365-385 is obscure. | Lines 365-385 | Lines 389-398 | We have clarified this section (to further support the additional clarification in response to replies 2.1 and 2.23) with additional text justifying the use of RRE, e.g. “for example in governing early decision making to maximise propagation yields” |
| 2.34 | What evidence do they have that transport stress is relevant to their nurseries at the study reef? What does RRE have to do with this statement? Isn’t this just another way to say, one site is better than the other? | Lines 365-385 | Lines 378 | The comment on transport stress has been removed from this sentence. |
| 2.35 | The authors state that local impacts (but they don’t identify what they mean by local impacts) could be minimized somehow by coral nurseries while at the same time boosting local coral abundance. Nothing in their study addressed outplanting. Nothing in their study addressed local impacts. | Conclusion | Line 309 and throughout | The specific sentence has been altered to reduce misinterpretation and readers drawing overstated conclusions.  We have made it clearer at the start of the ms that we are only focussing on the nursery phase of coral gardening.  Previous studies have shown the ability for nursery grown corals to be outplanted onto reefs, which was a core goal of this current work (although we specifically focus on the nursery phase for this current MS). As such, we have now modified the first paragraph of the discussion to clarify, “evaluate how low-cost nurseries could be used to propagate coral [26] to support high throughput outplanting [27]…” |
| 2.36 | The condition and growth of corals in nurseries if often better than seen in the wild, because the corals are up off the bottom in a more vigorous hydrodynamic environment. | Conclusion | N/A | Since we do not have accurate environmental data for each of our nursery sites, we do not wish to make assumptions on the cause of potentially higher growth rates in the nurseries as opposed to those seen in the wild. Especially given the lack of wild growth rates for coral taxa on the GBR. |
