# Supplementary material for: Coral growth, survivorship and return-on-effort within nurseries at high-value sites on the Great Barrier Reef
Source: PLoS One. 2021 Jan 11;16(1):e0244961. doi: 10.1371/journal.pone.0244961 (PMC7799815; doi:10.1371/journal.pone.0244961)
Supplement: Attachment — Submitted filename: Response to Reviewers 1.docx [file pone.0244961.s010.docx]

| Reviewer & comment no. | Reviewer comment | Line no. in original ms | Line no. in revised ms | Author comment |
| --- | --- | --- | --- | --- |
|  | We thank the Editor and Reviewer 3 for their positive, reflective and fair review of our manuscript. We appreciate their suggestions that we have incorporated them to improve our manuscript. | | | |
| Editor 1 | "complementing", not "complimenting" | Line 19 | Line 19 | Changed to “complementing” |
| E2 | Use plural – “translate” | Line 25 | Line 25 | Changed to “translate” |
| E3 | “complements”, not “compliments” | Line 82 | Line 82 | Changed to “complement” |
| E4 | “assessments of growth or survivorship” | Line 82 | Line 82 | Changed to “assessments of growth or survivorship” |
| E5 | “resulted in more fragments on the” | Line 163 | Line 163 | Changed to “resulted in more fragments on the” |
| E6 | suggest to change to "assessed for tracking growth" - the term "fate-tracking" likely led to the irritation by the previous reviewer #2 with this section | Line 166 | Line 166 | “fate-tracking” changed to “tracking” |
| E7 | Likewise suggest to simply use “tracked fragments” | Line 191 | Line 191 | “fate-tracked” changed to “tracked” |
| E8 | Slightly confusing sentence, suggest rephrasing to "but instead in terms of a potential life-history strategy trade-off" | Line 203 | Lines 203-205 | We agree that this sentence needed rephrasing. It was changed to: “In order to further evaluate success, we subsequently determined the relative growth rates as % growth month^-1^ (= [ΔG/G_I_] · 100), where G_I_ is initial size, in addition to assessing the life-history strategy trade-off of growth versus survivorship via RRE [26]” |
| E9 | “tracked fragments” | Lines 252 and 254 | Lines 252 and 254 | “fate-tracked” changed to “tracked” |
| E10 | Why is a significant test result reported if no differences were observed? Test result in Table S4 is "ns". | Line 296 | Line 296 | This was a typo. “p<0.001” changed to “p>0.05” |
| Reviewer 3.1 | Typo error for the word “propagated” | Line 6 | Line 6 | “propogated” changed to “propagated” |
| 3.2 | Change “rarely subject” to “rarely subjected” | Line 128 | Line 128 | “subject” changed to “subjected” |
| 3.3 | Don’t italicize “cf.” and make it consistent throughout the manuscript | Line 157 | Throughout | All instances of “cf.” are now non-italicized |
| 3.4 | Abbreviate A. tenuis | Line 163 | Line 162 | “*Acropora tenuis*” changed to “*A. tenuis*” |
| 3.5 | "...any fragments with visible signs of tissue loss were removed" does this mean that even if the tissue loss was very minimal (e.g. 10% loss) for a particular fragment, it was still removed? and what did you do to those fragments? can clarify this in text | Line 190 | Line 188 | Text currently states, “any fragments with visible signs of tissue loss were removed (as per permitting conditions to pre-empt potential disease outbreaks)”. We believe this statement doesn’t need any further clarification. Any tissue loss has the potential to lead to disease outbreaks within the nurseries, and removal of the fragments was a requirement of the permit. |
| 3.6 | Is this mainly to aid the Discussion? If so, then I suggest mentioning this in the Discussion instead, when comparing your results to previous studies | Line 209 | Line 209 | This point was not elaborated on in the Discussion as we did not observe any dependency of growth rate on initial fragment size. The comparison to previous studies has been removed from the Results. |
| 3.7 | I suggest to briefly mention how to interpret RRE scores | Line 214 | Line 215 | This statement was added: “with higher RRE scores indicating higher ΔG and survivorship” |
| 3.8 | I suggest not to abbreviate those species from different genus but begin with the same letter i.e. Porites cylindrica and Pocillopora cf. verrucosa | Line 234 | Line 234 and throughout | These abbreviations have been left in the text as this is consistent with previous studies. We believe there is little chance of confusion between genera given the unique species names. |
| 3.9 | Insert ‘space’ between A. and humilis | Line 296 | Line 294 | Space has been added. |
| 3.10 | Should be written as P. cf verrucosa throughout the manuscript, not P. verrucosa | Line 303 | Throughout | All instances of “*P. verrucosa*” have been changed to “*P.* cf *verrucosa*” |
| 3.11 | Can provide a range or a percentage loss of coral cover? and do you mean the entire GBR? | Line 312 | Line 310 | The percentage loss of coral cover on the GBR has been given in the Introduction (see lines 38-39). “at GBR sites” has been changed to “on the GBR” for clarification, and an additional reference has been added. |
| 3.12 | I suggest to split the statements. Separate the statement - "Notably, Biongiorni et al. ......" | Lines 321-325 | Lines 319-323 | These statements have been separated. |
| 3.13 | Can you discuss briefly as to why corals exhibited higher growth rates in areas next to high flow channel? can mention other studies that examined the effect of hydrodynamics on coral growth? | Line 344 | Lines 339-340 | This statement was added: “where higher flow rates have been reported to drive an increase in active feeding of corals whilst simultaneously reducing predation rates by corallivorous fish [35]”. Reference [35] has been added to the manuscript to provide further information on this topic. |
| 3.14 | Typo error for the word “comprehensive” | Line 390 | Line 386 | “compensive” changed to “comprehensive” |
| 3.15 | Add the word 'interpreted' between "should be" and "with caution" | Line 398 | Line 394 | Changed to “should be interpreted with caution” |
| 3.16 | Journal name should be 'Marine Pollution Bulletin' not 'Marine pollution bulletin' | Reference 1 | Reference 1 | Reference changed. |
| 3.17 | Journal name should be 'Bulletin of Marine Science' not 'Bulletin of marine science' | Reference 5 | Reference 5 | Reference changed. |
| 3.18 | Journal name should be 'Restoration Ecology' not Restoration ecology' | Reference 8 | Reference 8 | Reference changed. |
| 3.19 | Italicize Acropora cervicornis | References 10, 17 & 35 | References 10, 17 & 36 | Reference changed. |
| 3.20 | Journal name should be 'Ecological Engineering' not 'Ecological engineering' | Reference 21 | Reference 21 | Reference changed. |
| 3.21 | Journal name should be 'BMC Bioinformatics' not 'BMC bioinformatics' | Reference 29 | Reference 29 | Reference changed. |
| 3.22 | Italicize Acropora spp. | Reference 34 | Reference 34 | Reference changed. |
| 3.23 | Journal name should be 'Scientific Reports' not 'Scientific reports' | Reference 37 | Reference 38 | Reference changed. |
